# Automated High-Content Live Animal Drug Screening Using *C. elegans* Expressing the Aggregation Prone Serpin α1-antitrypsin Z

**DOI:** 10.1371/journal.pone.0015460

**Published:** 2010-11-12

**Authors:** Sager J. Gosai, Joon Hyeok Kwak, Cliff J. Luke, Olivia S. Long, Dale E. King, Kevin J. Kovatch, Paul A. Johnston, Tong Ying Shun, John S. Lazo, David H. Perlmutter, Gary A. Silverman, Stephen C. Pak

**Affiliations:** 1 Department of Pediatrics, Cell Biology and Physiology, University of Pittsburgh School of Medicine, Children's Hospital of Pittsburgh of UPMC and Magee-Womens Hospital Research Institute, Pittsburgh, Pennsylvania, United States of America; 2 Department of Pharmacology and Chemical Biology, University of Pittsburgh Drug Discovery Institute, University of Pittsburgh, Pennsylvania, United States of America; Brown University, United States of America

## Abstract

The development of preclinical models amenable to live animal bioactive compound screening is an attractive approach to discovering effective pharmacological therapies for disorders caused by misfolded and aggregation-prone proteins. In general, however, live animal drug screening is labor and resource intensive, and has been hampered by the lack of robust assay designs and high throughput work-flows. Based on their small size, tissue transparency and ease of cultivation, the use of *C. elegans* should obviate many of the technical impediments associated with live animal drug screening. Moreover, their genetic tractability and accomplished record for providing insights into the molecular and cellular basis of human disease, should make *C. elegans* an ideal model system for *in vivo* drug discovery campaigns. The goal of this study was to determine whether *C. elegans* could be adapted to high-throughput and high-content drug screening strategies analogous to those developed for cell-based systems. Using transgenic animals expressing fluorescently-tagged proteins, we first developed a high-quality, high-throughput work-flow utilizing an automated fluorescence microscopy platform with integrated image acquisition and data analysis modules to qualitatively assess different biological processes including, growth, tissue development, cell viability and autophagy. We next adapted this technology to conduct a small molecule screen and identified compounds that altered the intracellular accumulation of the human aggregation prone mutant that causes liver disease in α1-antitrypsin deficiency. This study provides powerful validation for advancement in preclinical drug discovery campaigns by screening live *C. elegans* modeling α1-antitrypsin deficiency and other complex disease phenotypes on high-content imaging platforms.

## Introduction

The pathologic accumulation of misfolded or aggregation-prone proteins underlies a wide range of human diseases including neurodegenerative disorders (e.g., Alzheimer's disease, Huntington's disease, Parkinson's disease, amyotrophic lateral sclerosis, frontotemporal dementia, and spongiform encephalopathies), systemic amyloidoses (e.g., immunoglobulin light chain (AL), serum amyloid A (AA) and transthyretin (ATTR) amyloidosis), retinal dystrophies (e.g., non-syndromic forms of retinitis pigmentosa) and the serpinopathies (e.g., α1-antitrypsin (AT, SERPINA1) deficiency) [1], [2], [3], [4], [5]. Although a network of factors and pathways attempt to maintain protein homeostasis (proteostasis) by balancing bulk protein synthesis with proper folding, trafficking and turnover [6]; the gradual accretion of toxic oligomers and possibly higher order polymers or aggregates leads to cellular injury and death [7]. Moreover, many disease states, such as diabetes, malignancies, cardiovascular disease, systemic inflammation, sepsis, and aging also add stress to the proteostasis network, which contributes to organ dysfunction and exacerbation of the underlying disease states [6].

Notwithstanding their prevalence, effective therapies for protein misfolding disorders are lacking [1], [8], [9]. However, experimental studies show that genetic or pharmacologic enhancement of the proteostasis network reduces the accumulation of aggregation prone proteins [10], [11]. Thus, one approach to developing novel therapies capable of treating a wide-range of protein misfolding disorders is to conduct target-directed (reverse chemical genetic) screens for compounds that enhance the activity of the proteostasis network. The development of small-molecule therapeutics by target-directed strategies has been accelerating due to the genome-driven discovery of new drug targets, the expansion of natural and synthetic combinatorial chemistry compound collections and the development of high- and ultra high-throughput screening (HTS) technologies [12], [13]. Despite these advances, a lead series painstakingly developed *in vitro* may be abandoned due to a lack of activity or an unfavorable therapeutic index upon testing in mammalian cell cultures, vertebrate animals or phase 1 clinical trials [14], [15]. Frequently, attrition of a lead series is due to unfavorable drug absorption, distribution, metabolism, excretion or toxicity (ADMET) [16], [17].

Some ADMET deficiencies are avoided, by conducting the initial drug screens in cells, and numerous cell-based assay technologies have been developed for HTS lead generation [18], [19], [20], [21], [22], [23], [24], [25], [26]. The emergence of imaging platforms, which combine automated fluorescence image acquisition with quantitative cellular image analysis, has converted cell-based screening from simple assays measuring a single parameter into high-content screening (HCS) strategies assessing multiple information-rich parameters (e.g., size, shape, granularity and fluorescence intensity) for each cell in culture [18], [19], [20], [21], [22], [23], [24], [25], [26]. Temporal and spatial integration of these parameters facilitates the evaluation of compound effects on complex physiological processes such as cell death activation, cell-to-cell contacts, vesicular trafficking and the translocation of fluorescent markers to different subcellular locations [18], [19], [20], [21], [22], [23], [24], [25], [26].

While HCS using cell-based assays facilitate the rejection of compounds that are directly cytotoxic, they are unable to identify those that lack the desired therapeutic effect *in vivo*, or demonstrate deleterious side effects on complex developmental or physiological processes, such as cellular migration or synaptic transmission, respectively. Moreover, the therapeutic effects of compounds on the systemic aspects of protein misfolding disorders cannot be modeled easily in cell culture systems. For this reason, forward chemical genetic (i.e., phenotype-directed) screens using live animals that model human protein misfolding disease phenotypes might serve as suitable alternatives to target-directed reverse chemical screens [27]. Drug screens using live organisms provide several distinct advantages over molecular- or cell-based assays and include: 1) the assessment of ADMET characteristics at the earliest stages of the drug discovery process, 2) the identification of leads without detailed knowledge of specific disease-related targets or molecular pathways and 3) the avoidance of ascertainment biases associated with targeting pathways or molecules whose involvement may ultimately prove to be tangential to the disease process. Despite these advantages, the assimilation of live animals into drug screening protocols presents logistical challenges. These barriers include labor- and cost-intensive development of disease phenotypes; screening protocols that are low-throughput and unamenable to statistically robust HTS-like formats; and the prohibitive consumption of compound libraries. Over the last several years, however, investigators began adapting small organisms, such as *Caenorhabditis elegans* and *Danio rerio*, to HTS protocols [28], [29], [30], [31], [32], [33], [34], [35], [36]. Taken together, these studies suggest that organisms dispensed by automated liquid-handling workstations and cultivated in microtiter plates may provide an economical alternative to molecular and cell-based screens. *C. elegans*, in particular, should be an ideal candidate for live animal HCS campaigns, as their tissues are transparent at all developmental stages, the use of fluorescent probes and tissue-specific fluorescent transgenic markers to study physiological processes *in vivo* are well established, fundamental cellular processes are highly conserved across species, and aspects of mammalian diseases can be successfully modeled in these invertebrates (reviewed in [37], [38], [39], [40]). Nonetheless, experimental variables that affect high-quality HCS protocols, such as sample preparation, assay strategy, image acquisition and image analysis, have yet to be optimized for any organism [41]. The goal of this study was to develop an all-liquid work-flow strategy that eliminates a major bottleneck in the screening process and fully exploits the advantages of *C. elegans* as a platform for *in vivo* high-content and high-throughput pre-clinical drug discovery campaigns for protein misfolding disorders. Moreover, by adapting an automated system that streamlines the image acquisition and data analysis components to accurately define objects and detect tissue-specific changes using fluorescent markers, we can easily adapt this system to screen for compounds that modulate a wide range of normal physiological processes (e.g., growth, development, organogenesis and ageing) and pathological phenotypes (e.g., cell death pathways, neuromuscular degeneration, inborn errors of metabolism and host-pathogen interactions).

## Results

### Detection of *C. elegans* developmental stages based on size

**Figure 1:**
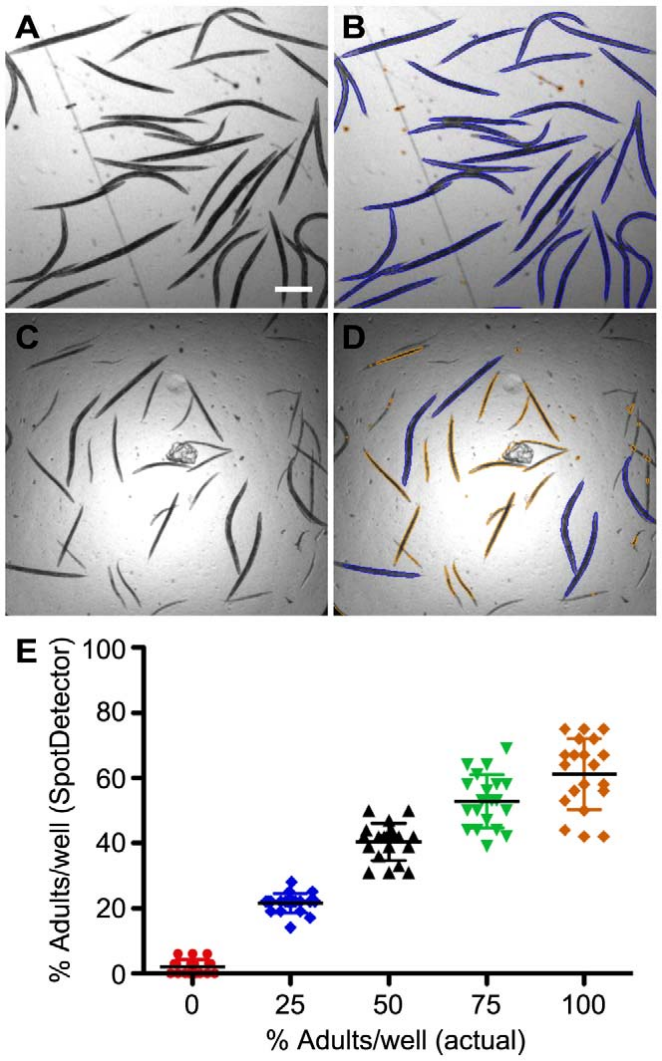
Animal (object) detection using the ArrayScan V^TI^. Thirty-six adult or mixed stage animals were dispensed into 384-well plates, imaged and analyzed using the ArrayScan V^TI^ and SpotDetector BioApplication. (A) A brightfield image of adult animals. (B) SpotDetector correctly identified all the worms in the field as indicated by the blue outline. (C) A representative brightfield image of a well containing 36 animals with a predetermined percentage (0, 25, 50, 75 and 100%) of adults sorted into a 384-well plate. (D) SpotDetector was optimized to identify large (L4 and adult stage) worms (blue outline) and exclude smaller (L1, L2 and L3 stage) worms (orange outline). (E) Correlation between the percent of adults actually sorted per well in (D) vs. the percent of adults as determined by SpotDetector. The slope and goodness-of-fit (*r* ^2^) of the linear regression were 0.72 and 0.85, respectively. The slope of the line was significantly different to 1 (*P*<0.05). Scale bar, 450 µm.

The inability to automate the image capture and data analysis steps continues to serve as the most significant bottleneck in live animal screening. In collaboration with Cellomics Inc., we adapted an automated fluorescence microscopy imaging system, originally designed for HCS and analysis of cultured cells (http://www.cellomics.com/content/menu/ArrayScan/), to automate the detection and analysis of *C. elegans* in a 96- or 384-well microtiter format. The instrument, ArrayScan V^TI^, consists of an inverted light microscope (Axiovert 200M, Carl Zeiss) configured with a motorized objective turret with Plan-Neofluar objectives, a motorized 5-position filter cube turret, a mechanized stage, a 12-bit cooled CCD camera and controller software. Samples are illuminated for brightfield imaging using a broad white-light source and for fluorescence imaging in up to 4 different spectra using a mercury-based light source. Different types of analysis modules (Thermo Scientific BioApplications) automatically convert 16-bit monochromatic images into numeric data. To determine whether the ArrayScan V^TI^ and the BioApplications software could accurately segment the images to identify and count small animals, we first assessed the number of young adult *C. elegans* sorted into a 384-well plate (Figure 1A). The software application required that objects first be defined and counted in channel 1. Using brightfield illumination, the SpotDetector BioApplication, which was programmed to detect dark objects on a bright background with a specified morphological size (width, length, area), identified nearly all the adult animals (Figure 1B, outlined in blue). Since the algorithm also excludes objects on the basis of size, we determined whether, the system could distinguish young adult animals from eggs and the smaller L1 through L4 larval forms. Populations of 36 animals, each containing different percentages of adult worms were sorted into wells of a 384-well microtiter plate (Figure 1C). The SpotDetector BioApplication correctly selected objects within (outlined in blue) and excluded objects without (outlined in yellow) the pre-selected size parameters (Figure 1D). However, in wells containing a higher proportion of adults, some animals were not counted. Miscounting, which decreased the overall goodness-of-fit of linear regression, was due to the inability of algorithm to resolve overlapping patterns into more than one discrete object (Figure 1E). As expected, the accuracy of detection improved when <10 adults were added to a well. Of note, the BioApplication can be configured to detect animals at, for example, the L1–L2 stage and exclude those at the L3–L4 adult stages (not shown). Taken together, these studies suggested that the instrument could be used to screen for compounds that alter the growth and development of synchronized cultures by counting the proportion of animals of a particular size at a constant time point.

### Detection of tissues, pathologic subcellular protein aggregates and autophagy within *C. elegans*

**Figure 2:**
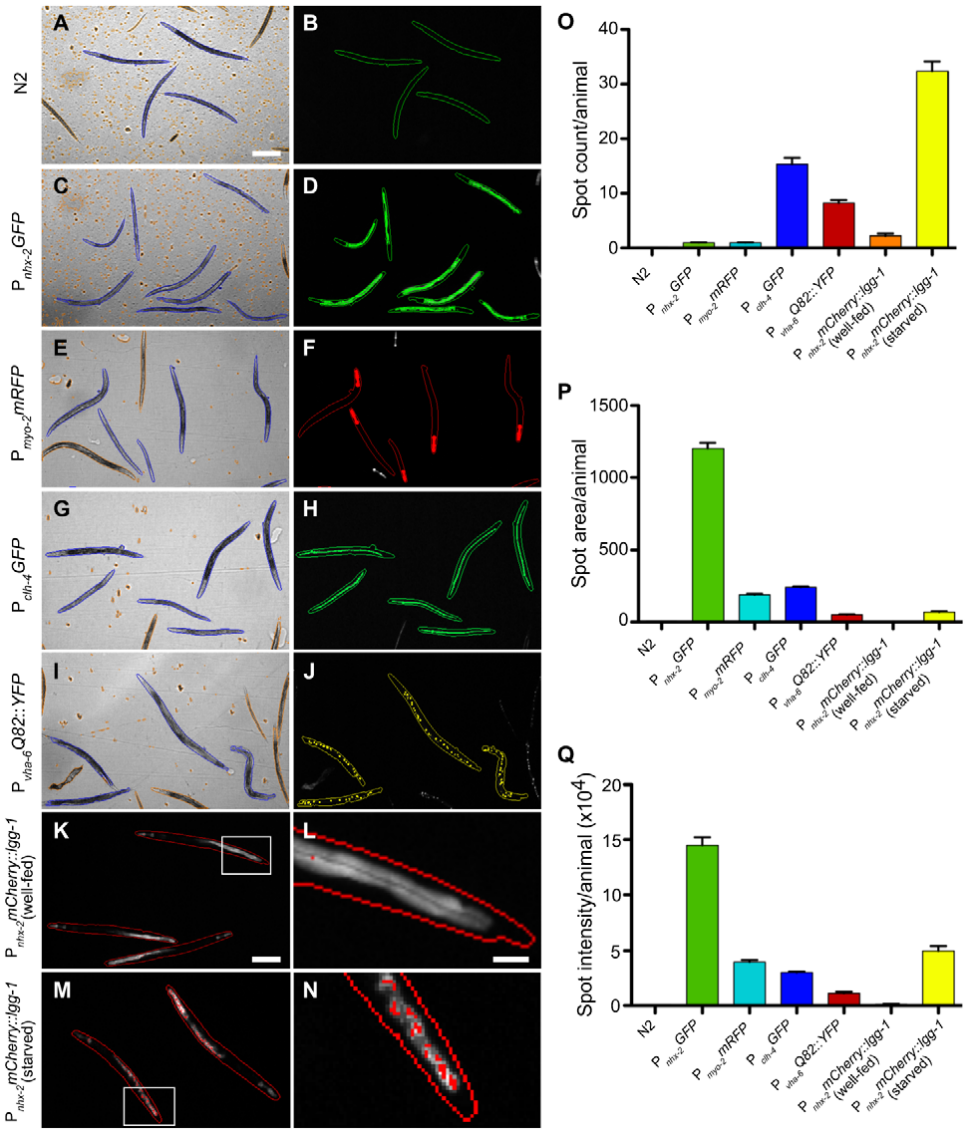
Automated detection and quantification of cells, tissues, subcellular protein aggregates or autophagy in individual animals. (A–J) The SpotDetector BioApplication was used to identify and quantitate different types of transgene expression (left of panels) in adult animals. The brightfield channel (left panels) was used to discriminate between complete adult animals (outlined in blue) and debris or incomplete animals (outlined in orange), while a fluorescence channel (colored overlays in right panels) was used to detect different types of fluorescently tagged transgenes in correctly identified objects. (K–N) Fluorescence images of well-fed (K) and starved (M) animals expressing the autophagy marker, mCherry::LGG-1. In well-fed animals, mCherry::LGG-1 was diffusely cytoplasmic (K). In contrast, induction of autophagy by starvation leads to a punctate fluorescence pattern within intestinal cells, as LGG-1 is incorporated in to autophagosomes (M). (L, N) Higher magnification of the boxed areas in (K) and (M), respectively. (O–Q) The different types of transgene expression were quantified by spot count (O), spot area (P) or spot intensity (Q) per animal. Spot count, spot area and spot intensity values for each of the transgenic lines were significantly (Student's *t*-test, *P*<0.001) different to that of N2 animals. Data derived from 10–50 wells containing ∼20 animals/well. Scale bars, 225 µm (A–J, K, M), 50 µm (L, N).

Once valid objects are selected using the brightfield images in channel 1, the ArrayScan V^TI^ can detect fluorescent “spots” in up to 4 different channels within each object and the SpotDetector BioApplication can display the data as a total fluorescent spot number, spot area or spot intensity per object. We next determined whether this application was sensitive enough to identify different cell types (pharyngeal cells, excretory cell, and intestinal cells), pathologic protein deposition (polyQ aggregates) or a physiological process (autophagy) within individual objects (animals). Fluorescent images (channel 2) were obtained for *C. elegans* strains carrying transgenes with tissue-specific promoters driving fluorescent protein expression in the pharynx (P*_myo-2_mRFP*), the excretory cell (P*_clh-4_GFP*) or intestinal cells (P*_vha-6_Q82::YFP*, P*_nhx-2_GFP* or P*_nhx-2_mCherry::lgg-1*). Except for the polyQ82-containing construct, which generates cytosolic aggregates [42], the others yielded a diffuse cytoplasmic fluorescence pattern under baseline conditions (Figure 2A–J). In comparison to the minimal background fluorescence of wild-type (N2) animals, the total spot number, area or fluorescence intensity per animal outputs of the SpotDetector BioApplication were markedly increased in transgenic animals (Figure 2O–Q). Depending on the nature of the transgene expression pattern, certain comparisons were more meaningful. For example, total spot area or total spot intensity per animal, rather than total spot count, were better at discriminating pharyngeal or intestinal expression in comparison to background (Figure 2C–F, P, Q). In contrast, total spot count per animal, was the more sensitive parameter to follow when assessing the presence of the excretory cell and the degree of protein aggregation in the animals expressing polyQ82 (Figure 2G–J, O).

Macroautophagy is a cellular process in which a double membrane envelops cytosolic components or organelles (autophagosome) and delivers this material to a lysosome (autophagolysosome) for degradation and recycling (reviewed in [43]). LGG-1/LC3/Atg8 is used as a marker for autophagosomes because it inserts specifically into the membranes of these vesicles as they are formed [44]. Upon autophagosome formation, LGG-1 fused to mCherry changes its cytoplasmic distribution pattern from diffuse (lower fluorescence intensity) to punctate (higher fluorescence intensity) [44]. To determine whether the imaging system could follow this process, we examined a strain expressing a P*_nhx-2_mCherry::lgg-1* transgene after starvation, a potent inducer of intestinal autophagosome formation [45]. In well-fed animals, the diffuse cytoplasmic fluorescence in the intestinal cells was well above that of the N2 background (Figure 2K–L, O–Q). To detect mCherry::LGG-1 puncta, the diffuse fluorescence intensity of the well-fed animals was used to calibrate and establish a threshold, above which the SpotDetector BioApplication would identify as high-intensity spots. Although basal autophagy in the well-fed animals yielded a few high-intensity spots (Figure 2O), the large number of distinct puncta in the starved animals (Figure 2M–N, O–Q) indicated a marked increase in autophagy that was detected best by a statistically significant increase in spot count or total spot intensity per animal (Figure 2O and Q, respectively). Taken together, this versatile imaging platform quantitatively measured several different types of fluorescence patterns, thereby allowing for the interrogation of a wider range of biological processes, such as tissue organization, proteotoxicity and metabolic functions.

### Detection of live cells and dead animals

**Figure 3:**
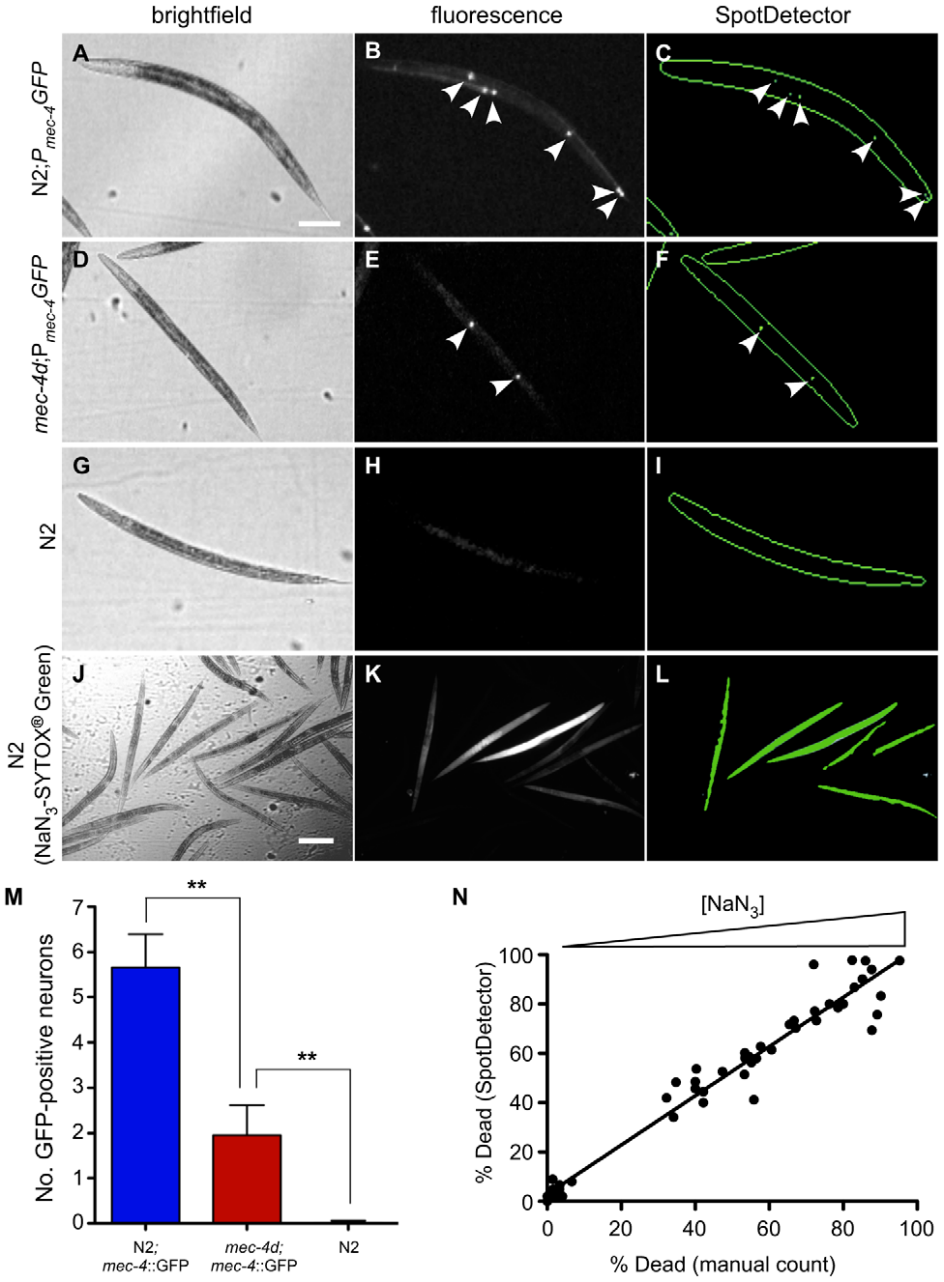
Identification of live cells or dead animals using *C. elegans*. The ArrayScan V^TI^ and SpotDetector BioApplication was used to discriminate between wild-type and toxic gain-of-function *mec-4(d)* mutants based on the survival of the 6 mechanosensory neurons in *C. elegans*. Brightfield (left), fluorescence (center) and SpotDetector rendered (right) images are depicted for each line. (A–C, M) In N2 (wild-type) animals, P*_mec-4_*GFP expression was evident within 5.7±0.7 touch-sensing neurons (arrowheads). (D–F, M) In the *mec-4(d)* mutant background, the number of P*_mec-4_*GFP expressing neurons (arrowheads) was significantly reduced and averaged 2.0±0.7 neurons per animal. (G–I, M) No GFP-positive neurons were identified in non-transgenic, N2 worms. Data derived from minimum of 32 wells containing ∼20 animals/well. Statistical significance determined using the Student's *t*-test, **P<0.001. The system was then used to discriminate live from dead animals. (J–L) Adult worms expressing the pharyngeal marker, P*_myo-2_*mRFP, were incubated with various concentrations of NaN_3_, stained with *SYTOX*® *Green* and imaged using the ArrayScan V^TI^ (J–K). The SpotDetector BioApplication was optimized to determine the percentage of dead animals by counting the number of *SYTOX*® *Green* -positive bodies (L) and dividing by the total number of P*_myo-2_*mRFP-positive heads (not shown) detected in the GFP and TRITC fluorescence channels, respectively. (N) Percentage of dead animals at different NaN_3_ concentrations as determined by visual inspection versus that determined by SpotDetector. The slope and goodness-of-fit (*r* ^2^) of the linear regression were 1.0 and 0.95, respectively. The slope of the line was not significantly different to 1 indicating near 1∶1 correlation (*P*>0.95). Scale bars, 100 µm (A–I), 225 µm, (J–L).

The nematode has served as an informative system to study the genetics of different modes of cell death. We examined whether this imaging system could distinguish between live or dead cells using either the loss or gain of a fluorescent marker, respectively. *mec-4*, a member of the DEG/ENaC membrane cation channel superfamily, is expressed exclusively in the 6 mechanosensory neurons of *C. elegans* [46]. A reporter strain containing an integrated transgene, ZB164 *bzIs8*[P*_mec-4_GFP*]; *mec-4(+)*, driving GFP expression in the mechanosensory neurons exhibits ∼4–5 fluorescent cell bodies per L4/young adult animal [47]. In contrast, post-developmental necrotic cell death gradually occurs in most of the mechanosensory neurons after the reporter strain is crossed with animals containing a toxic gain-of-function mutation, *mec-4(d)*. To determine whether the imaging system could distinguish the wild-type from the *mec-4(d)* strain, we identified adult animals by brightfield illumination in channel 1 (Figure 3A, D, G), and for comparison, by fluorescence imaging to display the GFP-labeled mechanosensory neurons in channel 2 (Figure 3B, E, H). SpotDetector quantified the number of live florescent cells (spots) present in each brightfield object (Figure 3C, F, I). Consistent with previous studies, the *mec-4(+)* and *mec-4(d)* strains averaged ∼6 and ∼2 cells/animal, respectively (Figure 3M) [47]. Remarkably, the system was capable of discriminating between wild-type and mutant animals based on the differential viability of just six mechanosensory neurons.

Animals exposed to toxic doses of sodium azide (NaN_3_) undergo massive necrotic intestinal cell death characterized by a marked loss of membrane permeability [48]. Thus, the uptake of the membrane impermeant fluorescent nucleic acid dye, SYTOX® Green, serves as a dead cell indicator [49]. To determine whether the system could discriminate dead from live intestinal cells, we scanned and analyzed young adult animals exposed to different concentrations of NaN_3_ in the presence SYTOX® Green. Dead animals showed extensive uptake of SYTOX® Green that was accurately detected by the imaging system (Figure 3J–L), as was shown by a dose dependent increase in the number of dead animals that correlated with the number counted manually (Figure 3N). We concluded that this automated system was capable of detecting dead cells and should prove useful in developing HCS for drugs that modulate necrotic cell death.

### Development of a HCS protocol using *C. elegans*

**Figure 4:**
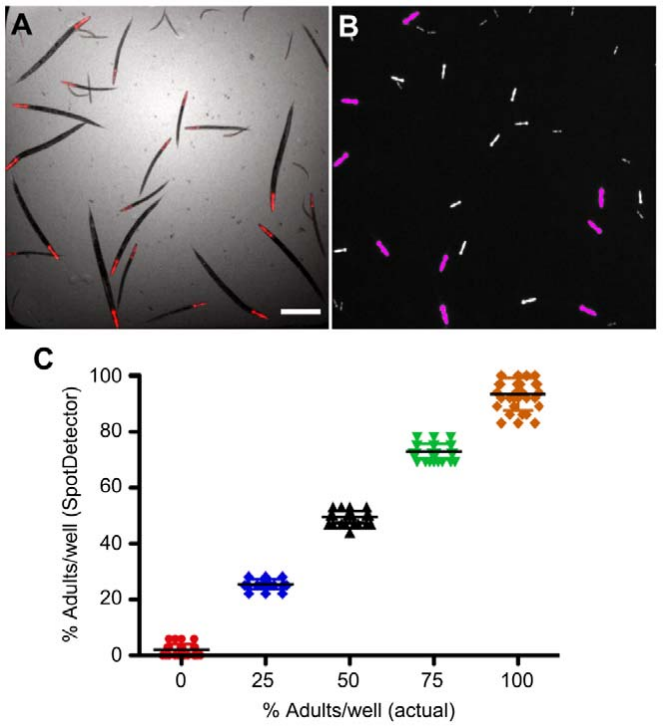
Identification of animals in a mixed population using a fluorescent head- marker. Thirty-six animals expressing the pharyngeal marker, P*_myo-2_*mRFP were sorted into wells of 384-well plate. The wells contained different percentages (0–100%) of L4/young, and the SpotDetector BioApplication was optimized to select this group and reject younger animals (L1, L2and L3 stages). (A) A brightfield-mRFP composite image of transgenic worms at different stages expressing P_myo-2_mRFP. (B) A SpotDetector image showing the ability to differentiate adults (magenta overlay) from earlier staged larvae (white overlay) based on a combination of fluorescent spot area and intensity in the pharyngeal region. (C) Correlation between the percent of adults actually sorted per well vs. the percent of adults as determined by SpotDetector. The slope and goodness-of-fit (*r* ^2^) of the linear regression were 0.92 and 1.0, respectively. The slope of the line was not significantly different to 1 indicating near 1∶1 correlation (*P*>0.05). Scale bar, 450 µm.

Although brightfield imaging in channel 1 accurately detected adult animals (objects) in the well of a 384-well plate (Figure 1), the time required to autofocus and capture each animal, plus a need to limit the adult worm population to ∼10 animals per well (due to overlapping) decreased throughput and assay robustness. To obviate these problems, we took advantage of the P*_myo-2_mRFP* transgenic animals that expressed the fluorescent protein in their pharyngeal region (Figure 2F). Since the total fluorescence area or total fluorescence intensity of this region was proportional to the overall size and developmental stage of the animals, we determined whether fluorescence imaging of the “red-heads” using these parameters could be substituted for the more time-consuming brightfield imaging. As above (Figure 1), we sorted populations of 36 transgenic animals, each containing different percentages of adult worms, into the wells of a 384-well microtiter plate. A composite brightfield and mRFP fluorescence image showed that all of the animals had a detectable red-head that was proportional in area to the developmental stage and size of the animal (Figure 4A). Next, we preset the brightfield optics in channel 1 to detect the entire well as a single “object”. Once an object was defined in channel 1, the SpotDetector BioApplication was programmed to select (pseudocolored red heads) or exclude (pseudocolored white heads) fluorescent spots above or below, respectively, a pre-determined threshold value based on a combination of fluorescent spot area and intensity (Figure 4B). In this example, the algorithm correctly identified all 9 young adult animals and excluded ∼24 of the larval forms (Figure 4B). Since, the area of the red-heads was proportionally smaller than that of adult animals, the total count was rarely confounded by overlapping pharyngeal “spots”. Thus, there was excellent correlation between the number of adult animals detected by the spot count image analysis parameter and the actual number of animals in the wells (Figure 4C). We concluded that the number of adult animals accurately detected in a well of a 384-well plate increased from ∼10 to at least 35, when fluorescence imaging of the red-head marker, rather than the brightfield imaging of individual animals was used to obtain a valid animal count.

**Figure 5:**
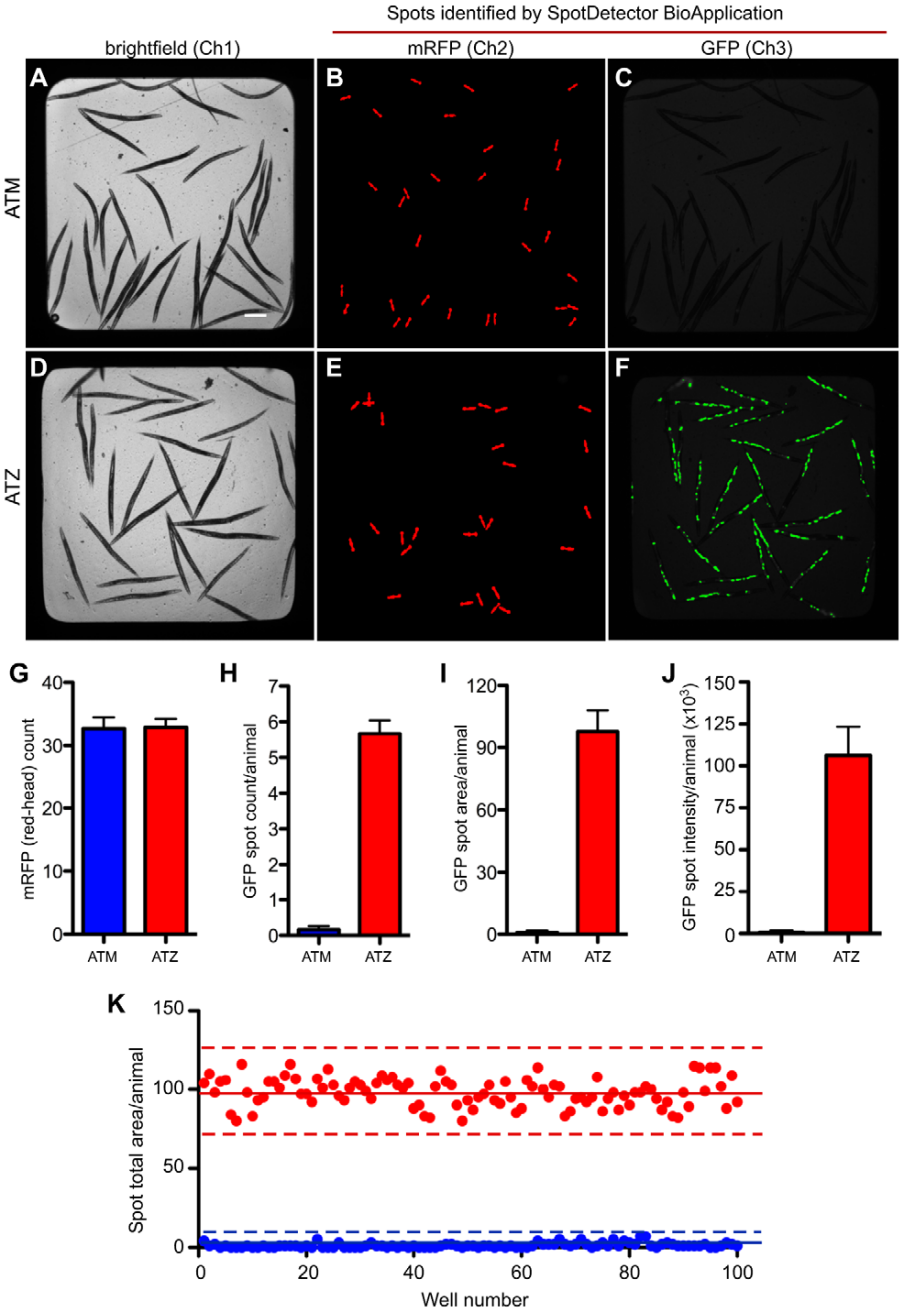
High-content analysis of transgenic animals expressing the wild-type (ATM) and mutant (ATZ) forms of human α1-antitrypsin (AT) fused to GFP. Thirty-five young adult animals were sorted into wells of a 385-well plate and imaged using the ArrayScan V^TI^. (A, D) Brightfield images of sGFP::ATM and sGFP::ATZ expressing transgenic animals, respectively. (B, E) SpotDetector images of fluorescent red heads for corresponding transgenic lines pictured in (A) and (D), respectively. (C, F) SpotDetector images of sGFP::ATM and sGFP::ATZ expressing transgenic animals imaged in (B) and (E), respectively. (G) The average number of transgenic animals in each well was determined by counting the number of mRFP-positive heads in channel 2 (TRITC). (H–J) The amount of sGFP::ATZ (green intracellular inclusions) accumulating within the intestinal cells of transgenic animals was compared to that of the sGFP::ATM line using the SpotDetector BioApplication to analyze the signal detected in channel 3 (GFP). Animals expressing the mutant protein (ATZ) were distinguished clearly from those animals expressing the wild-type protein (ATM) whether comparing total spot count (H), area (I) or intensity (J) per animal. Number of animals analyzed 2,240 (ATM) and 2,240 (ATZ). Error bars represent SD. (K) Assay quality was assessed using a scatter plot comparing total GFP-spot area/well (n = 100 wells or 3,500 animals per strain) of sGFP::ATZ animals (red dots) to that of wild-type animals (blue dots). Solid and dotted lines indicate the mean spot area±3 standard deviations from the mean, respectively. The Z′-factor for this assay≈0.7.

Since the imaging system can detect fluorescent “spots” in more than one channel, we next determined whether a combination of two different fluorescent markers could be used to develop a high-content drug screening strategy using live animals. First, we developed an integrated transgenic line expressing the Z-mutation of the human secreted serpin, α1-antitrypsin (ATZ). This transgene, P*_nhx2_sGFP::ATZ*, contains a human ATZ minigene fused C-terminal to GFP with an N-terminal signal peptide (sGFP) (Pak et al., manuscript in preparation). An intestinal-specific promoter from *nhx-2* was used to drive fusion gene expression [50]. In humans, this common Z mutation induces protein misfolding and accumulation within the endoplasmic reticulum of hepatocytes resulting in cellular injury and cirrhosis (reviewed in [51]). Similarly, sGFP::ATZ aggregated within the endoplasmic reticulum of intestinal cells. As a control, we also generated an integrated transgenic line, expressing the wild-type fusion protein, sGFP::ATM. This protein was efficiently secreted into the intestinal lumen and pseudocoelomic space and was detectable microscopically only after a relatively long integration time (Pak et al., manuscript in preparation). To facilitate analysis using the ArrayScan V^TI^, both strains were co-injected with the P*_myo-2_mRFP* transgene. Approximately 35 animals expressing sGFP::ATZ or sGFP::ATM were sorted into 384-well plates. To minimize variability, only P*_nhx2_sGFP::ATZ* animals within a tight fluorescence window were sorted into the wells. The entire well was imaged in channel 1 using brightfield illumination (Figure 5A, D). These images, which were not used to identify individual animals, simply confirmed that comparable numbers of young adult animals of both lines were sorted into the wells. Using channel 2 and 3, respectively, SpotDetector identified the red-heads (Figure 5B, E) and either sGFP::ATM (barely detectable at the integration time used, Figure 5C) or sGFP::ATZ (Figure 5F) expression in the two different transgenic lines. Next, the red-heads detected in channel 2 were used to determine a “head count” and to show that the actual number of animals sorted into each well were nearly identical (Figure 5G). Finally, the images obtained in channel 3 were used to measure three different parameters in each of the wells containing sGFP::ATM or sGFP::ATZ expressing animals (Figure 5H–J). The total GFP spot number, area or intensity divided by the head count (i.e., parameter average per animal) were significantly increased in the sGFP::ATZ animals as compared to those of the sGFP::ATM expressing animals (Figure 5H–J). Indeed, at the integration time used, sGFP::ATM expression was not significantly above that of wild-type animals (not shown).

Compared to the control animals, we concluded that the steady-state amounts of sGFP::ATZ in the transgenic line provided a dynamic range amenable to screening for compounds that alter sGFP::ATZ accumulation. However, prior to initiating a HCS campaign, we tested the overall quality of the assay, using the Z′-factor as a metric [52]. The Z′-factor coefficient, which is calculated from the mean and the SD of the negative and positive control populations, is an indicator of HTS assay quality, robustness and reproducibility. Values between 0.5 and 1 are considered excellent and highly desirable prerequisite before conducting a HTS/HCS campaign. To determine the quality of this assay, 200 wells containing either sGFP::ATM or sGFP::ATZ animals were imaged using the ArrayScan V^TI^. In a representative experiment, the mean total spot area per sGFP::ATM and sGFP::ATZ animals were 1.3±1.5 and 98.1±9.0, respectively (Figure 5K). The Z′-factor for this assay was ∼0.7. Within a single experiment (sort) the Z′-factor remained constant from plate-to-plate. However, the Z′-factor varied as much as 0.4 to 0.7 from day-to-day depending mostly on the size of the sort-window used to select the P*_nhx2_sGFP::ATZ* animals (not shown).

### Compound screen

**Figure 6:**
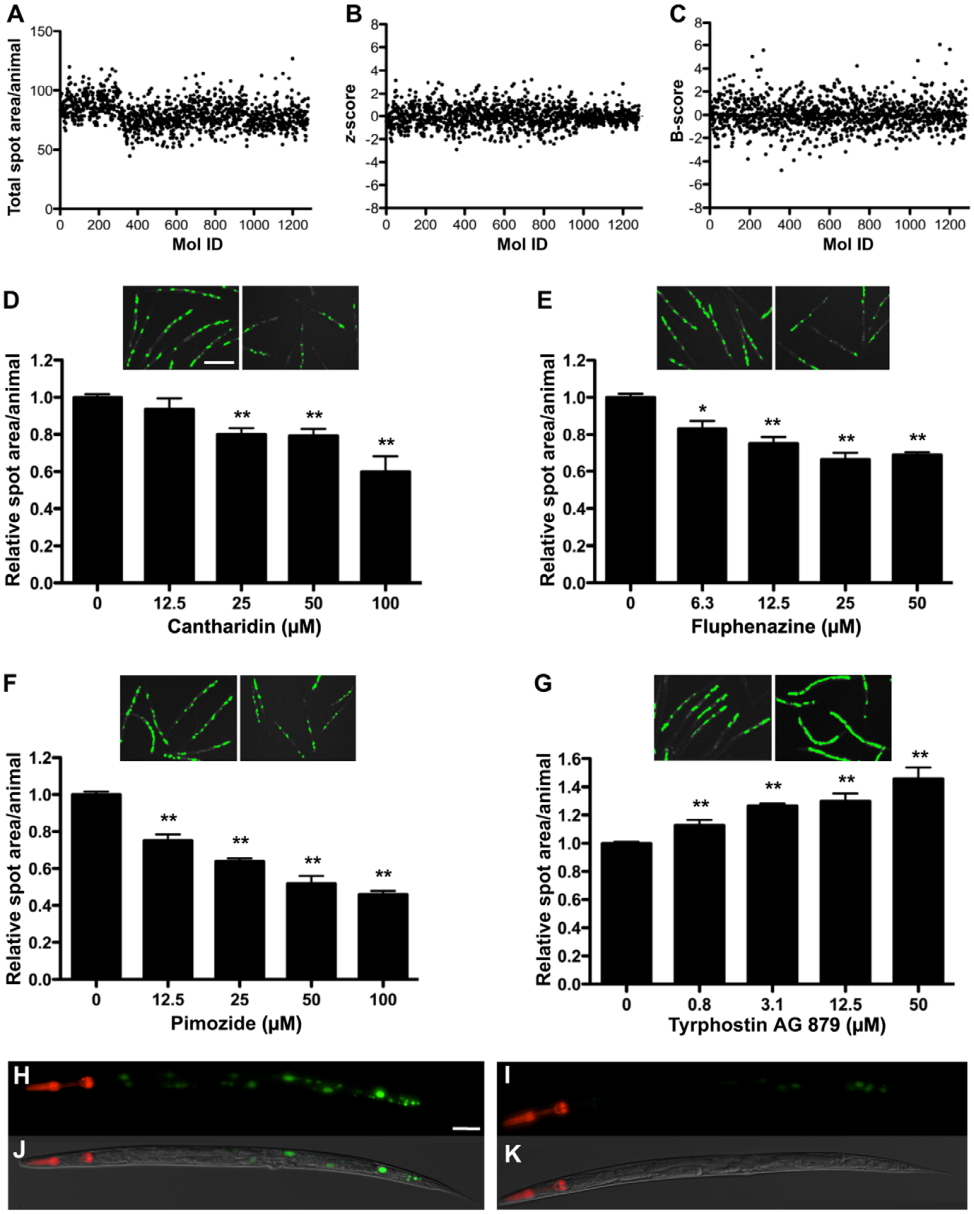
LOPAC library screen. (A) Total spot area per animal (object), (B) *z*-scores and (C) B-scores from a representative screen to measure the effects of 1280 LOPAC compounds on sGFP::ATZ accumulation in transgenic animals. The x-axis represents the molecular identification (Mol ID) number of the compound. Known autofluorescent compounds were excluded from the plot. Selected compounds, based on rank-order (Table 1) were analyzed for dose-dependent responses. Well images and dose-responses were obtained for compounds that decreased ((D) cantharidin, (E) fluphenazine and (F) pimozide) or increased ((G) tyrphostin AG 879) sGFP::ATZ accumulation. In each panel (D–G), well images on the left and right are DMSO (control)- and drug-treated animals, respectively. (H–K) Higher magnification fluorescent (top) and merged DIC (bottom) images of (H, J) DMSO- or (I, K) cantharidin- treated animals. Note loss of GFP::ATZ accumulation in the cantharidin treated animal. Scale bars, 450 µm (D–G) and 50 µm (H–K). Error bars represent SEM. Number of animals used was 140 for each compound concentration and 520 for the DMSO control. Significance was determined using an unpaired Student's *t*-test. Asterisks indicate values that differed significantly from animals treated with DMSO. *P<0.01 and **P<0.001.

**Figure 7:**
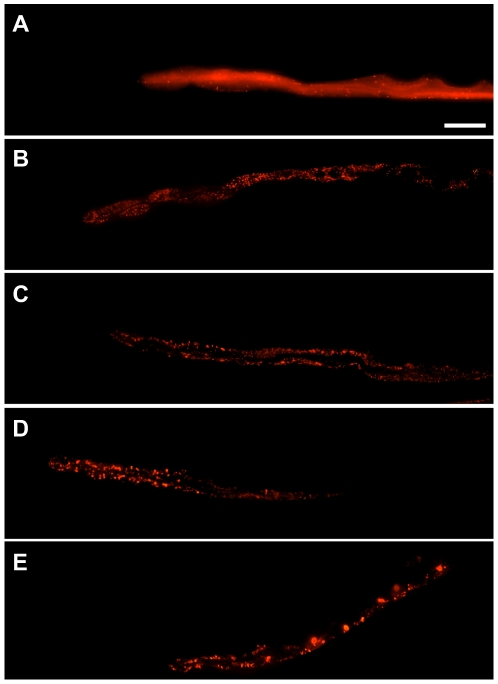
Induction of autophagy by hit compounds. Images of transgenic animals expressing P*_nhx-2_mCherry_::_lgg-1* treated with various compounds are shown. Images were acquired using a Nikon instruments Ti*Eclipse* widefield light microscope fitted with a 20× Plan Apochromat objective. Images were deconvolved using Volocity (Perkin Elmer, v 5.3.2). Deconvolved z planes were merged to a single plane. Well-fed animals treated with (A) DMSO show a diffuse mCherry expression throughout the intestine. In contrast, animals treated with (B) cantharidin, (C) fluphenazine and (D) pimozide show a markedly punctate distribution pattern indicative of increased autophagic activity. (E) Starved animals are included as a positive control for autophagy. Scale bar, 50 µm.

To test the HCS protocol, we performed a pilot drug screen using the library of pharmacologically active compounds (LOPAC1280™, 1280 compounds). Tight gating parameters for total fluorescence were used to sort 35 young adult P*_nhx2_sGFP::ATZ* animals into wells of 384-well plates containing 50 µM of a LOPAC compound and 0.5% DMSO. P*_nhx2_sGFP::ATM* and P*_nhx2_sGFP::ATZ* animals incubated with 0.5% DMSO served as untreated controls and were placed in the first-two and last-two columns of each plate. In a representative experiment, plate 1 of the LOPAC library was set-up for screening on day 1, and 3 other plates were set-up on the next day. After a 24 hour incubation at 22°C, animals were immobilized by the addition of NaN_3_ and placed in the ArrayScan V^TI^ for automated imaging. To examine for systematic errors, the raw data (total spot area/animal) were depicted as a plate-well scatter plot (Figure 6A). From these data, a small amount of drift was seen in plate 1 in comparison to plates 2–4. This difference reflects a slight variation in the sort-window used to collect animals on day 1 in comparison to that used on day 2. As the average values of the negative controls and that of the sample wells were similar, we combined the control and sample wells for normalization and to identify potential hits using the *z*-score (Figure 6B). The ArrayScan V^TI^ reads microtiter plates by rows, alternating from left-to-right, and then right-to-left. For some assays, we noted that the control fluorescence values would drift upwards slightly in rows towards the bottom of the plate. This drift appeared to correlate with an increase in chamber temperature during the scanning period and was minimized by cooling the chamber with a fan or shortening the read times by using 2.5× objective with a 0.63× coupler. Nonetheless, we controlled for intra-plate variation by presenting the data as a B-score (Figure 6C). Under the same conditions, we repeated the entire screen on a single day. We created an average rank-score for each compound by first compiling a list for each screen based on ascending B-scores, and then calculating the average rank for each compound (Table 1). To verify potential hits, we arbitrarily focused on those compounds with rank-scores <110 (n = 33) or >1225 (n = 15). Generally, compounds with these rank-scores had B-scores lesser or greater than 3 in at least one of the screens, and demonstrated the ability to significantly decrease or increase sGFP::ATZ accumulation, respectively. Based on cost and commercial availability, we selected 16 compounds to test for dose-dependent effects (Table 1). Cantharidin (Figure 6D), fluphenazine (Figure 6E) and pimozide (Figure 6F) were representative examples of 6 of 12 compounds that showed a dose-dependent decrease in sGFP::ATZ accumulation; whereas tyrphostin (Figure 6G) was an example of 3 of 4 compounds that showed an increase in sGFP::ATZ accumulation. Interestingly, all three compounds that decreased GFP::ATZ accumulation were isolated previously in screens for compounds that enhance autophagy, a known elimination pathway for ATZ. When we treated animals expressing the P*_nhx-2_mCherry::lgg-1* transgene with cantharidin, fluphenazine and pimozide, the distribution of mCherry::LGG-1 changed from diffusely cytosolic to punctate, suggesting an increase in the number of autophagosomes (Figure 7). To ensure that the puncta were not due to drug-induced mCherry aggregation, we treated animals expressing a P*_nhx-2_mCherry* transgene with fluphenazine. No mCherry puncta were detected (Figure S1). Taken together, these studies suggest that this screening assay was capable of identifying hit compounds that significantly altered sGFP::ATZ accumulation.

**Table 1:** Potential Hit Compounds that alter sGFP::ATZ accumulation.

Rank-score^a^	Overall rank-order^b^	Potential hit compound
Compounds that decreased ATZ accumulation:		
2.0	1	ivermectin^d^
2.5	2	cantharidin^c^
10.5	3	L-655,240
24.0	4	GR 125487 sulfamate salt
28.0	5	muscimol hydrobromide
34.5	6	DL-homatropine hydrobromide
36.5	7	L(-)-norepinephrine bitartrate^d^
41.5	8	N-(2-[4-(4-Chlorophenyl)piperazin-1-yl]ethyl)-3-methoxybenzamide
51.0	9	cefmetazole sodium^d^
52.5	10	HA-100
56.0	11	SB 206553 hydrochloride
57.5	12	L-701,324
57.5	13	phenamil methanesulfonate^d^
58.0	14	rolipram
61.0	15	doxepin hydrochloride^d^
61.5	16	beta-chloro-L-alanine hydrochloride
65.0	17	S(-)-UH-301 hydrochloride
72.5	18	L-alpha-methyl DOPA
73.5	19	taxol^c^
74.0	20	cis-(Z)-flupenthixol dihydrochloride
75.5	21	10-(alpha-diethylaminopropionyl)-phenothiazine hydrochloride
79.0	22	cantharidic acid^c^
81.0	23	fluphenazine dihydrochloride^c^
83.5	24	tamoxifen citrate^c^
85.0	25	indirubin-3′-oxime
89.5	26	(−)-bicuculline methbromide, 1(S), 9(R)
90.0	27	cephradine
93.0	28	indatraline hydrochloride
95.5	29	5-carboxamidotryptamine maleate
98.0	30	tyrphostin AG 112
103.5	31	prochlorperazine dimaleate
105.5	32	B-HT 933 dihydrochloride^d^
107.5	33	pimozide^c^
Compounds that increased ATZ accumulation:		
1263.0	1	GW2974
1256.0	2	thapsigargin^c^
1255.0	3	SB 224289 hydrochloride
1255.0	4	clotrimazole
1253.5	5	IC 261
1245.5	6	tetradecylthioacetic acid
1245.0	7	tyrphostin 1
1240.5	8	(+)-bromocriptine methanesulfonate
1238.0	9	L-162,313
1236.5	10	tyrphostin AG 879^c^
1236.5	11	IIK7
1234.0	12	glipizide^d^
1233.5	12	WIN 62,577
1231.0	14	(R)-(+)-WIN 55,212-2 mesylate
1229.5	15	rottlerin^c^

a Rank-scores were calculated by averaging compound rankings based on ascending B-scores from two independent drug screens. Compounds with rank-scores <110 or >1225 significantly decreased or increased the accumulation of sGFP::ATZ inclusions, respectively.

b Overall rank-order, based on relative rank-scores, for compounds that decreased or increased sGFP::ATZ accumulation.

c Compound demonstrated a dose-dependent response.

d Compound failed to demonstrate a dose-dependent response.

## Discussion

As a platform for drug discovery, *C. elegans* provides several distinct advantages over other *in vivo* models. Well established reverse and forward genetic screening technologies permits target validation or target identification of new hit compounds selected by forward chemical screening campaigns. Their small size and ability to grow in liquid medium makes them amenable to high-throughput work-flows using large-particle (animal) sorters, higher order microtiter plates and liquid handling robotic work-stations. Their transparency at all developmental stages and ease of creating transgenic lines makes them ideal for studying a range of biological processes using fluorescence microscopy. Finally, live animal screens appear to be ideal candidates for drug discovery strategies designed to address ADMET deficiencies at the outset of the screening process [53]. In practical terms, however, two major obstacles block the adaptation of small animals, such as *C. elegans*, to high-throughput, high-content screening protocols: the absence of 1) high-quality assays and 2) an automated system to capture, analyze and store data documenting the biological effects of thousands of compounds [41]. Using a combination of transgenic lines expressing fluorescent proteins and a commercially available automated fluorescence microscopy imaging system, the ArrayScan V^TI^, we showed that the *C. elegans* was capable of serving as a high-quality drug discovery platform analogous to those devised for cell-based HCS protocols [19].

To improve assay quality we focused initially on parameters that affected sample population variability. Despite using integrated and staged transgenic lines, the fluorescence intensity of the sGFP::ATZ-expressing animals varied two-fold. We minimized this variability in the assay population by using the COPAS™ *BIOSORT* to collect a precise number of animals using a tightly-gated size and fluorescence intensity window. The growth conditions in the microtiter wells also had a profound effect on assay quality. *C. elegans* Maintenance Medium (CeMM, chemically defined, bacteria free medium) appeared to be an ideal growth medium for animals cultivated in microtiter plates, but intense autofluorescence precluded further use [54]. We ultimately used S Medium supplemented with antibiotics and *E. coli* (OP50). Antibiotics were included to prevent growth of fast-growing bacterial contaminates that could negatively affect survival of the nematodes. Defining the optimal growth conditions, which vary depending on the length of the assay period and the number and condition of the animals, were crucial to developing a robust and reproducible assay.

The second major impediment to the routine use of *C. elegans* in HCS was the lack of systems that automated the time-consuming process of image acquisition, analysis and storage. This bottleneck is evident in the first series of relatively low-throughput and labor-intensive *C. elegans* drug screens [28], [29], [33], [55], [56]. Compound effects were assessed by direct inspection of animals in microtiter plate wells using a stereomicroscope or of images captured by a CCD camera. Although sensitive for the detection of certain phenotypes, such as alterations in movement or morphology, manual inspection of plates or images is time consuming and tedious for HTS campaigns scaled for assaying hundreds-of-thousands of compounds [28], [29], [33]. Moreover, operator fatigue increases variability and decreases specificity. An enzymatic assay that measures fluorescent substrate conversion in culture medium can be automated, but the effects of compounds on the whole animal are lost [55]. Recently, Moy et al., reported an automated high-throughput screen for novel antimicrobial compounds that protect *C. elegans* from a lethal dose of *S. faecalis* [31]. While their automated screening assay was five-times faster than screening manually, the algorithm was limited to a simple yes-no (live-dead) assessment using the uptake of SYTOX® Orange as an indicator of death, and was unable to quantify the effects of compounds on individual animals. Taken together and as compared to established cell-based HCS protocols, these studies suggest that whole animal HCS was cumbersome and lacked the refinements in image acquisition and analysis to quantitatively assess compound effects on continuous physiological variables such as growth and development, autophagy, misfolded protein disposition and cell permeability. In contrast, the HCS format we developed captured images from up to 5 channels of each well of a 384-well microtiter plate using an automated inverted fluorescence microscope platform, while the image analysis software converted these signals into multiparametric output in real time. Since the images were stored on a server, different algorithms could be applied at different times to extract various quantitative measures, such as fluorescent spot count, spot area or spot intensity per animal. These types of qualitative measures could never be assessed accurately manually, as the time required to count, for example, a dozen fluorescent spots in 35 animals in each well of a 384-well plate would rapidly fatigue even the most fastidious observer. However, we recommend that the stored images still be scanned occasionally for overall quality control and to examine outliers for irregularities that might account for a large change in signal. For example, several wells with high total fluorescence were due to autofluorescent compounds rather than hits that enhanced the expression or accumulation of sGFP::ATZ. Similarly, for wells in which the fluorescence was diminished, by for example 50%, inspection of the images allowed us to distinguish between those compounds that decreased sGFP::ATZ accumulation comparably in all animals versus those that simply killed half the animals without affecting the signal from the others. The ability to eliminate nuisance compounds at the earliest stages of the screening process was a significant advantage to using this imaging system.

The instrument settings used for image acquisition also affected assay quality, and were improved considerably by optimizing the microscope's autofocus and scanning times, and the degree of magnification used to scan the wells of 96- versus 384-well plates. The analysis algorithms, which were used to establish the fluorescence intensity cut-offs and to define fluorescent objects, also had a significant impact on overall assay quality. By adjusting these parameters, we consistently obtained a Z′-factor, which serves as a measure of assay quality, in the excellent range of 0.5 to 0.7; scores that rivaled those of the highest quality cell- or molecule-based HTS schemes [52]. Based on our studies using transgenic animals expressing misfolded sGFP::ATZ, we concluded that the ArrayScan V^TI^ and the BioApplication programs possessed the automation, speed and sensitivity to generate a high-quality assay that would permit the quantitative assessment of compound libraries on a continuous physiological variable, such as misfolded protein accumulation. Moreover, by optimizing the imaging analysis, we reduced the scanning time of a 384-well plate to ∼30 minutes. Thus, ∼6,000 compounds could be screened in a typical workday, or ∼18,000 compounds per day if the ArrayScan V^TI^ was configured with an automated plate loader. Screening of compound effects on a discrete variable, such as a live-dead screen, would be even faster.

As a test of our *C. elegans* screening strategy, we performed a limited drug screen for compounds that affect accumulation of the misfolded human serpin, α1-antitrypin (sGFP::ATZ). Of the 6 compounds inducing a concentration-dependent decrease in sGFP::ATZ accumulation, four (cantharidin, tamoxifen, fluphenazine and pimozide) belong to classes of drugs that were identified previously as enhancers of autophagy [57], [58]—a physiological process involved in the elimination of ATZ [59]. Thus, the drug discovery strategy outlined in this report has significant clinical import, as *C. elegans* has been used to model several protein misfolding disorders including Alzheimer's disease [60], frontotemporal dementia with parkinsonism chromosome 17 type [61], Parkinson's disease [62], polyglutamine repeat disorders [63] and amyotrophic lateral sclerosis [64]. Taken together, we suggest that live animal HCS for compounds that ameliorate disorders of proteostasis is feasible [6]. [6], [60], [63].

## Materials and Methods

### Construction of promoter-transgene fusions

We constructed a transcriptional P*_myo-2_mRFP* fusion construct was by subcloning the *myo-2* promoter and the mRFP cDNA into the SphI/XbaI and NheI/EcoRV sites of the canonical expression vector, pPD49.26 (a kind gift from Dr. Andrew Fire, Stanford University School of Medicine), respectively. To generate the P*_nhx-2_mCherry::lgg-1* construct, we PCR amplified a 3.5 kb genomic fragment containing the *lgg-1* promoter, coding region and 3′-UTR and cloned it into pCR®-Blunt II-TOPO® vector (Invitrogen, Carlsbad, CA, USA). Using site directed mutagenesis a unique MluI restriction enzyme site, was introduced upstream of the *lgg-1* translation start codon. The mCherry cDNA, lacking a translation stop codon, was inserted into the MluI site, which places it in-frame with the *lgg-1* coding region. To direct expression of the *mCherry::lgg-1* fusion gene in intestinal cells, we replaced the *lgg-1* promoter with a 1.5 kb *nhx-2* promoter using a HindIII restriction site.

P*_nhx-2_sGFP::ATM* was generated by inserting a 1.5 kb *nhx-2* promoter fragment into HindIII/XbaI restriction sites of the expression vector, pPD95.85. We then introduced a KasI restriction site by site-directed mutagenesis into the GFP translational stop codon. A 1.4 kb fragment containing the ATM cDNA and 3 synthetic introns was then cloned into the KasI site. P*_nhx-2_sGFP::ATZ* was generated by site-directed mutagenesis of P*_nhx-2_sGFP::ATM*, thereby generating the E342K (Z) mutation.

The plasmid containing P*_nhx-2_GFP*, pFH6II*nhx-2*, was a kind gift from Keith Nehrke (University of Rochester Medical Center) [65].

### Worm strain and culture conditions

Worm strains: VK413 (P*_nhx-2_GFP*), VK1093 (P*_nhx-2_mCherry::lgg-1*), VK821 (P*_myo-2_mRFP*) were generated by injecting the respective plasmids into the gonad of young adult N2 hermaphrodites at a final concentration 80 ng/µl. Strains VK689 (P*_nhx-2_sGFP::ATM*) and VK694 (P*_nhx-2_sGFP::ATZ*) were generated by co-injecting the plasmids and P*_myo-2_mRFP* at a final concentration of 70 ng/ml and 10 ng/ml, respectively. The worm strain expressing P*_clh-4_GFP* (pFL6II*clh-4*) were a gift from Keith Nehrke [65]. N2 and GF66 (P*_vha-4_Q82::YFP*, [42]) were obtained from *Caenorhabditis* Genetics Center (CGC), http://www.cbs.umn.edu/CGC/). Worms were routinely cultured at 22°C on nematode growth medium (NGM) plates seeded with *E. coli* strain, OP50, unless otherwise specified.

### Imaging of transgenic animals using ArrayScan V^TI^

Twenty N2 or transgenic L4-adult stage worms were transferred to 384-well plates containing 60 µl of PBS and anesthetized with 30 µl of 0.02 M NaN_3_ prior to image capture. NaN_3_ treatment prevents z-axis movements ensuring animals are in a uniform plane for imaging. Images were acquired with the ArrayScan V^TI^ HCS Reader (Cellomics, ThermoFisher, Pittsburgh, PA, USA) fitted with a 5× or 2.5× objective and a 0.63× coupler. For the detection of various developmental stages using N2 worms, images were captured using the brightfield channel. Valid objects (adult worms) were automatically selected using the SpotDetector BioApplication (Cellomics). For image capture and analysis of the lines expressing fluorescent transgenes, we employed a 2-channel (brightfield and GFP or TRITC) assay. Algorithms were optimized to first identify valid objects (blue outline in Figs.), defined as non-overlapping, whole worms in the brightfield channel. Debris and partial worms were automatically excluded (orange outline in Figs.) from analysis. Fluorescent transgene expression, within valid objects, was quantified in the TRITC or GFP channels. SpotDetector BioApplication was optimized to identify transgene expression as spots. Parameters were optimized such that spots of varying shape, size and intensity could be identified. For this paper, spot count, spot total area and spot total intensity per object were used to compare transgene expression in different animals.

### Whole animal alive-dead assay

Adult N2;*vkIs1033[P_myo-2_::mRFP]* animals were incubated at room temperature with sodium azide (0–100 mM) for 4 hours. Animals were washed 5 times with M9 medium and stained with 2 µM SYTOX™ Green (Invitrogen) for 5 minutes at room temperature [48]. Approximately 50 animals/well were dispensed into an optical bottom black walled 96 well plate (Nunc Thermo Fisher Scientific, Rochester, NY, USA). Wells were imaged using the ArrayScan V^TI^ over the entire area of the well in brightfield, red (TRITC) and green (GFP) channels at 50× magnification. The total number of animals and the number of dead bodies were determined by counting red and green spots, respectively. Data from SpotDetector algorithm were confirmed by manual counting of the live and dead animals. Percent dead = (the number of green objects detected / total number of animals)×100.

### OP50 preparation for growth of animals in liquid culture

A single colony of OP50 was placed in 3 ml LB broth and incubated at 37°C with vigorous shaking overnight. One milliliter of overnight culture was added to 1 L sterile LB broth and was incubated at 37°C with vigorous shaking until reaching an OD_600_ = 0.5. The bacteria were washed twice with PBS and concentrated to an OD_600_ = 10.0. An equal volume of 50% glycerol was added for long-term storage at −80°C. After thawing, the bacteria were concentrated by centrifugation and re-suspended in PBS to an OD_600_ = 10.0.

### Preparation of animals for HCS drug screening

Ten adult animals were placed on twelve 10 cm plates of NGM agar medium spread with a lawn of *E. coli* strain OP50 (NGM/OP50). Approximately 7 days later, young adult stage F2 animals were isolated by differential sedimentation and transferred to 12 NGM/OP50 plates. After an overnight incubation at 22°C, adults were washed off with PBS and the remaining eggs were allowed to hatch overnight. Early-stage larvae were transferred to 48 NGM/OP50 plates and allowed to grow until most of the worms were in the 4^th^ larval (L4) stage. Using the COPAS™ *BIOSORT* (Union Biometrica, Holliston, MA, USA) approximately 15,000 L4 stage animals expressing similar levels of GFP were sorted into twelve 10 cm NGM/OP50 plates. After an overnight incubation at 22°C, gravid adults were washed off and transferred to fresh NGM/OP50 plates and allowed to lay eggs for 5 hours. Following this incubation period, adults were washed off and discarded leaving a synchronous population of eggs on the plates. The eggs were incubated at 22°C for 16 hours. The hatchlings were then washed off and transferred to 80–100 100 mm NGM plates seeded with OP50. After further incubation for 28–32 hours or until the majority of the worms were in the L4/young adult stages. This method generated a population of ∼200,000 age-synchronized animals for small molecule screening.

In preparation for sorting, animals were washed off plates and transferred into 50 ml conical tubes and allowed to settle by gravity for 5 minutes. After discarding the supernatant, animals were washed again with 50 ml of PBS to remove excess bacteria and other debris that could interfere with worm sorting. Following the final rinse, total worm count was determined by taking aliquots of the worm suspension. The final worm concentration was routinely adjusted to ∼400 animals/ml.

### Compound Libraries and handling, dilution and transfer to assay plates

The 1280 compound Library of Pharmacologically Active Compounds (LOPAC) was purchased from Sigma-Aldrich (St. Louis, MO, USA). Compounds were arrayed into 384-well microtiter master plates at a concentration of 10 mM in DMSO. LOPAC compounds were given unique University of Pittsburgh Drug Discovery Institute (UPDDI) substance identity numbers and were handled and stored as described previously [21], [22], [23], [66]. Daughter plates containing 2 µl of 10 mM compounds in DMSO were prepared and replicated from the LOPAC master plates using the Vprep (Agilent Technologies, Santa Clara CA, USA) outfitted with a 384-well transfer head. Aluminum adhesive plate seals were applied with an Abgene Seal-IT 100 (Rochester, NY, USA) plate sealer and plates were stored at −20°C in a Matrical MatriMinistore™ (Spokane, WA, USA) automated compound storage and retrieval system. For the primary screen, daughter plates were withdrawn from the −20°C freezer, thawed at ambient temperature and centrifuged 1–2 min at 50×*g*. The plate seals were removed and 98 µl of S-medium were added to the wells using the Flex Drop dispenser (Perkin Elmer, Waltham, MA, USA). This intermediate stock of library compounds was at a concentration of 200 µM in 2% DMSO. The diluted compounds were mixed by repeated aspiration and dispensation using a 384-well P30 dispensing head on the Evolution-P3 (EP3) liquid handling platform (Perkin Elmer), and then 15 µl of each compound were transferred to the wells of assay plates. In the primary screen, compounds were screened individually at a final concentration of 50 µM.

### Assay plate preparation for drug screen

On the day of the screen, assay plates containing 15 µl of each compound were thawed and centrifuged at 214×*g* for 60 s. Fifteen microliters of 4× assay medium, which was prepared by mixing 4.0 ml OP50, 25.4 ml S-medium, 0.6 ml 100× antibiotic-antimycotic stock solution (stock contained 10,000 units penicillin, 10 mg streptomycin and 25 µg amphotericin B/ml, Sigma) and 120 µl 200 mM FUDR, were added to each well. Animals were then sorted into the wells using the COPAS™ *BIOSORT* worm sorter.

### Animal sorting using the COPAS™ *BIOSORT*

To reduce assay variability, a tightly-synchronized population of worms was selected based on size (i.e., stage of development) and fluorescence intensity (i.e., transgene expression) using the COPAS™ *BIOSORT*. L4 to young adult-stage worms were initially selected using empirically-determined time-of-flight (TOF) and coefficient of extinction (EXT) values. Animals were also gated based on GFP fluorescence intensity. Approximately 30% of the starting population was selected.

For analytical assays, animals were suspended in S-medium (minus EDTA) for sorting. The flow rate was maintained at ∼25 worms/sec. Coincidence check was employed to enhance selection specificity. For LOPAC library screening, COPAS sheath fluid was replaced with 0.01% Triton X-100 in S-medium (minus EDTA) to promote healthy bacteria and worm growth. Thirty-five L4 to young-adult animals were sorted into wells containing compounds and assay medium. The final total volume per well after addition of the animals was 60 µl. Approximately 45,000 worms were required for each 384-well plate. On average, sorting time was 90 minutes per plate. The flow cell was periodically flushed between plates to prevent clogging. Four 384-well plates were routinely sorted on the same day. Plates were then sealed with ThinSeal T-2417-4 (ISC BioExpress, Kaysville, UT, USA) and incubated at 22°C for 24–48 hours.

### Imaging of animals using the ArrayScan V^TI^

Prior to imaging, worms were anesthetized by adding 30 µl of 0.02 M NaN_3_ in PBS to each well. Plates were resealed, inverted twice, and incubated for 5 minutes at room temperature. Images were acquired with the ArrayScan V^TI^ HCS Reader fitted with a 2.5× objective and a 0.63× coupler using a 2-channel (TRITC and GFP) assay. Real-time analysis was performed using the SpotDetector BioApplication optimized to quantify fluorescent protein expression in *C. elegans*. Image acquisition and analysis of a 384-well plate was completed in <45 minutes.

The total number of animals in the well was determined by counting the number of red heads (P*_myo-_*_2_mRFP) in the TRITC channel. Total spot area or total spot intensity was determined by quantifying the GFP-positive spots in the GFP channel. Total spot area or total spot intensity per animal was determined by dividing the values from the GFP channel by that from the TRITC channel.

### HCS data analysis

Compound tracking and data analysis for the primary HCS assay were performed using ActivityBase™ (IDBS, Guildford, UK), CytoMiner (UPDDI) software and visualized using Spotfire™ DecisionSite® (TIBCO Software Inc., Somerville, MA, USA) software, as described previously [21], [22], [23], [66]. Custom calculators were written to process the HCS data and perform the z-score and B-score statistical analysis [67], [68].

As a measure of assay quality and robustness, we utilized the Z′-factor [52]. The Z′-factor was calculated from the mean and the standard deviation of the negative and positive control populations as follows:
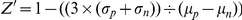
where *σ* is the standard deviation, *μ* is the mean and *p* and *n* are positive and negative controls, respectively. Z′-factors between 0.5 and 1.0 indicate the separation band (signal window) between the positive and negative controls is wide and the assay is of excellent quality and suitable for HTS/HCS. Z′-factors between 0 and 0.5 indicate a good quality screen, whereas a score <0 indicates the assay is of poor quality and unsuitable for HTS/HCS.

We utilized the *z*-score plate-based statistical scoring method as described previously to identify compounds that behaved as statistical outliers compared to the other substances (n = 320, no controls) tested on an assay plate for selected HCS multi-parameter measurements output by the image analysis module [22]. The *z*-score = (X_i_−,X.)/*σ*, where X_i_ was the raw measurement on the *i*_th_ compound, and ,X. and *σ* were the mean and standard deviation of all the sample measurements on a plate.

The B-score was calculated from all of the sample measurements on an assay plate and used an iterative mathematical model to eliminate systematic row and column artifacts on a plate. The mathematical model of the B-score was described as:
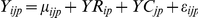
where 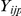 was the compound measurement at *i*_th_ row and *j*_th_ column of the *p*_th_ plate, 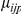 was the ‘true’ activity value, 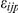 was the random error of the assay on the *p*_th_ plate, and 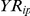 and 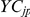 represented the row and column artifacts on the *p*_th_ plate, respectively. A two-way median polish statistic method was applied to estimate the B-score of a HCS assay. The implemented procedures are described below. The random error estimate, 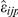, of the measurement at *i*_th_ row and *j*_th_ column of the *p*_th_ plate was calculated by fitting a two-way median polish as:
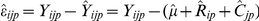
where 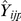 was the fitted compound value, 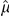 was the estimated average of the plate, and 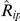 and 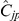 were the estimated systematic artifacts for the *i*_th_ row on *p*_th_ plate and *j*_th_ column on *p*_th_ plate, respectively. Next the median absolute deviation (MAD) of the random error estimate on *p*_th_ plate was computed as:
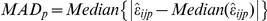
At the last step, the B score was calculated as:
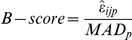



The compounds were ranked according to ascending B-score values. Rank-scores were calculated by taking the average of compound rankings from two independent drug screens. Compounds with rank-scores <110 significantly decreased the accumulation of sGFP::ATZ inclusions. Conversely, compounds with rank-scores >1225 significantly increased the accumulation of sGFP::ATZ inclusions. Selected compounds (based on cost and availability) from both groups were chosen for further analysis.

### Hit compound characterization

Compounds that were identified as potential hits were purchased (if available) and retested for verification. Compounds that failed to produce a dose-dependent response were not analyzed further. Compounds that produced a response in a dose-dependent manner were further tested for a time-dependent response.

Compound dose-response curves were performed by dispensing 15 µl of a 4× stock solution into 384-well plates containing 15 µl of assay medium (see above). Thirty-five animals were sorted into each well bringing the volume to ∼60 µl. The final compound concentrations in each well varied from 0–100 µM. Assay plates were incubated in a 22°C incubator for 24 or 48 hours. Each compound was tested in quadruplicate in at lease 2 independent experiments.

### Statistical evaluation

Statistical evaluation of data was performed using *Prism*® (Graphpad Software). The significance of actual and predicted data in Figures 1, 3 and 4 was determined using a linear regression analysis and comparing the slope and goodness-of-fit (*r* ^2^) values. Statistical significance of the spot count, spot area and spot intensity values between N2 (wild-type) and various transgenic lines in Figure 2 and dose-response in Figure 6 was determined using an unpaired, one-tailed, Student's *t*-test.

## Supporting Information

Figure S1 **Effect of fluphenazine on mCherry aggregation.** To determine whether fluphenazine causes non-specific aggregation of mCherry, transgenic animals expressing P*_nhx-2_Ub-M::mCherry* were exposed to fluphenazine for 24 hours. The *Ub-M::mCherry* fusion is used as a control for ubiquitin (Ub) fusion protein degradation. Upon Ub cleavage, the absence of a degradation signal (due to an N-terminal methionine) prevents mCherry degradation. Thus, mCherry behaves as an unmodified cytosolic protein [69]. P*_nhx-2_mCherry::lgg-1* (top) and P*_nhx-2_Ub(GM)::mCherry* (bottom) animals were treated with (A, C) 0.5% DMSO or (B, D) 50 µM fluphenazine. Images were acquired using a Nikon instruments Ti*Eclipse* widefield light microscope fitted with a 40× objective. Scale bar, 100 µm. (JPG)

## References

[1] Aguzzi A, O'Connor T (2010). Protein aggregation diseases: pathogenicity and therapeutic perspectives.. Nat Rev Drug Discov.

[2] Herczenik E, Gebbink MF (2008). Molecular and cellular aspects of protein misfolding and disease.. FASEB J.

[3] Krebs MP, Holden DC, Joshi P, Clark CL, Lee AH (2010). Molecular mechanisms of rhodopsin retinitis pigmentosa and the efficacy of pharmacological rescue.. J Mol Biol.

[4] Lomas DA, Belorgey D, Mallya M, Miranda E, Kinghorn KJ (2005). Molecular mousetraps and the serpinopathies.. Biochem Soc Trans.

[5] Picken MM (2010). Amyloidosis-where are we now and where are we heading?. Arch Pathol Lab Med.

[6] Balch WE, Morimoto RI, Dillin A, Kelly JW (2008). Adapting proteostasis for disease intervention.. Science.

[7] Cohen E, Bieschke J, Perciavalle RM, Kelly JW, Dillin A (2006). Opposing activities protect against age-onset proteotoxicity.. Science.

[8] Bartolini M, Andrisano V (2010). Strategies for the inhibition of protein aggregation in human diseases.. Chembiochem.

[9] Krainc D (2010). Clearance of mutant proteins as a therapeutic target in neurodegenerative diseases.. Arch Neurol.

[10] Hidvegi T, Ewing M, Hale P, Dippold C, Beckett C (2010). An autophagy-enhancing drug promotes degradation of mutant alpha1-antitrypsin Z and reduces hepatic fibrosis.. Science.

[11] Link CD (2006). C. elegans models of age-associated neurodegenerative diseases: lessons from transgenic worm models of Alzheimer's disease.. Exp Gerontol.

[12] Frearson JA, Collie IT (2009). HTS and hit finding in academia - from chemical genomics to drug discovery.. Drug Discov Today.

[13] Mayr LM, Bojanic D (2009). Novel trends in high-throughput screening.. Curr Opin Pharmacol.

[14] Bleicher KH, Bohm HJ, Muller K, Alanine AI (2003). Hit and lead generation: beyond high-throughput screening.. Nat Rev Drug Discov.

[15] Hodgson J (2001). ADMET–turning chemicals into drugs.. Nat Biotechnol.

[16] Gleeson MP (2008). Generation of a set of simple, interpretable ADMET rules of thumb.. J Med Chem.

[17] Gleeson P, Bravi G, Modi S, Lowe D (2009). ADMET rules of thumb II: A comparison of the effects of common substituents on a range of ADMET parameters.. Bioorg Med Chem.

[18] Giuliano KA, Johnston PA, Gough A, Taylor DL (2006). Systems cell biology based on high-content screening.. Methods Enzymol.

[19] Haney SA, LaPan P, Pan J, Zhang J (2006). High-content screening moves to the front of the line.. Drug Discov Today.

[20] Johnston PA (2002). Cellular platforms for HTS: three case studies.. Drug Discov Today.

[21] Johnston PA, Foster CA, Tierno MB, Shun TY, Shinde SN (2009). Cdc25B dual-specificity phosphatase inhibitors identified in a high-throughput screen of the NIH compound library.. Assay Drug Dev Technol.

[22] Johnston PA, Phillips J, Shun TY, Shinde S, Lazo JS (2007). HTS identifies novel and specific uncompetitive inhibitors of the two-component NS2B-NS3 proteinase of West Nile virus.. Assay Drug Dev Technol.

[23] Johnston PA, Soares KM, Shinde SN, Foster CA, Shun TY (2008). Development of a 384-well colorimetric assay to quantify hydrogen peroxide generated by the redox cycling of compounds in the presence of reducing agents.. Assay Drug Dev Technol.

[24] Nickischer D, Laethem C, Trask OJ, Williams RG, Kandasamy R (2006). Development and implementation of three mitogen-activated protein kinase (MAPK) signaling pathway imaging assays to provide MAPK module selectivity profiling for kinase inhibitors: MK2-EGFP translocation, c-Jun, and ERK activation.. Methods Enzymol.

[25] Trask OJ, Baker A, Williams RG, Nickischer D, Kandasamy R (2006). Assay development and case history of a 32K-biased library high-content MK2-EGFP translocation screen to identify p38 mitogen-activated protein kinase inhibitors on the ArrayScan 3.1 imaging platform.. Methods Enzymol.

[26] Williams RG, Kandasamy R, Nickischer D, Trask OJ, Laethem C (2006). Generation and characterization of a stable MK2-EGFP cell line and subsequent development of a high-content imaging assay on the Cellomics ArrayScan platform to screen for p38 mitogen-activated protein kinase inhibitors.. Methods Enzymol.

[27] Stockwell BR (2000). Chemical genetics: ligand-based discovery of gene function.. Nat Rev Genet.

[28] Breger J, Fuchs BB, Aperis G, Moy TI, Ausubel FM (2007). Antifungal chemical compounds identified using a C. elegans pathogenicity assay.. PLoS Pathog.

[29] Kwok TC, Ricker N, Fraser R, Chan AW, Burns A (2006). A small-molecule screen in C. elegans yields a new calcium channel antagonist.. Nature.

[30] Molina G, Vogt A, Bakan A, Dai W, Queiroz de Oliveira P (2009). Zebrafish chemical screening reveals an inhibitor of Dusp6 that expands cardiac cell lineages.. Nat Chem Biol.

[31] Moy TI, Conery AL, Larkins-Ford J, Wu G, Mazitschek R (2009). High-throughput screen for novel antimicrobials using a whole animal infection model.. ACS Chem Biol.

[32] North TE, Goessling W, Walkley CR, Lengerke C, Kopani KR (2007). Prostaglandin E2 regulates vertebrate haematopoietic stem cell homeostasis.. Nature.

[33] Petrascheck M, Ye X, Buck LB (2007). An antidepressant that extends lifespan in adult Caenorhabditis elegans.. Nature.

[34] Rihel J, Prober DA, Arvanites A, Lam K, Zimmerman S (2010). Zebrafish behavioral profiling links drugs to biological targets and rest/wake regulation.. Science.

[35] Tran TC, Sneed B, Haider J, Blavo D, White A (2007). Automated, quantitative screening assay for antiangiogenic compounds using transgenic zebrafish.. Cancer Res.

[36] Yu PB, Hong CC, Sachidanandan C, Babitt JL, Deng DY (2008). Dorsomorphin inhibits BMP signals required for embryogenesis and iron metabolism.. Nat Chem Biol.

[37] Artal-Sanz M, de Jong L, Tavernarakis N (2006). Caenorhabditis elegans: a versatile platform for drug discovery.. Biotechnol J.

[38] de Voer G, Peters D, Taschner PE (2008). Caenorhabditis elegans as a model for lysosomal storage disorders.. Biochim Biophys Acta.

[39] Kaletta T, Hengartner MO (2006). Finding function in novel targets: C. elegans as a model organism.. Nat Rev Drug Discov.

[40] Silverman GA, Luke CJ, Bhatia SR, Long OS, Vetica AC (2009). Modeling molecular and cellular aspects of human disease using the nematode Caenorhabditis elegans.. Pediatr Res.

[41] Lee S, Howell BJ (2006). High-content screening: emerging hardware and software technologies.. Methods Enzymol.

[42] Morley JF, Brignull HR, Weyers JJ, Morimoto RI (2002). The threshold for polyglutamine-expansion protein aggregation and cellular toxicity is dynamic and influenced by aging in Caenorhabditis elegans.. Proc Natl Acad Sci U S A.

[43] Lum JJ, DeBerardinis RJ, Thompson CB (2005). Autophagy in metazoans: cell survival in the land of plenty.. Nat Rev Mol Cell Biol.

[44] Melendez A, Talloczy Z, Seaman M, Eskelinen EL, Hall DH (2003). Autophagy genes are essential for dauer development and life-span extension in C. elegans.. Science.

[45] Kang C, You YJ, Avery L (2007). Dual roles of autophagy in the survival of Caenorhabditis elegans during starvation.. Genes Dev.

[46] Driscoll M, Chalfie M (1991). The mec-4 gene is a member of a family of Caenorhabditis elegans genes that can mutate to induce neuronal degeneration.. Nature.

[47] Royal DC, Bianchi L, Royal MA, Lizzio M, Mukherjee G (2005). Temperature-sensitive mutant of the Caenorhabditis elegans neurotoxic MEC-4(d) DEG/ENaC channel identifies a site required for trafficking or surface maintenance.. J Biol Chem.

[48] Luke CJ, Pak SC, Askew YS, Naviglia TL, Askew DJ (2007). An intracellular serpin regulates necrosis by inhibiting the induction and sequelae of lysosomal injury.. Cell.

[49] Gill MS, Olsen A, Sampayo JN, Lithgow GJ (2003). An automated high-throughput assay for survival of the nematode Caenorhabditis elegans.. Free Radic Biol Med.

[50] Nehrke K (2003). A reduction in intestinal cell pHi due to loss of the Caenorhabditis elegans Na+/H+ exchanger NHX-2 increases life span.. J Biol Chem.

[51] Perlmutter DH (2002). Liver injury in alpha1-antitrypsin deficiency: an aggregated protein induces mitochondrial injury.. Journal of Clinical Investigation.

[52] Zhang JH, Chung TD, Oldenburg KR (1999). A Simple Statistical Parameter for Use in Evaluation and Validation of High Throughput Screening Assays.. J Biomol Screen.

[53] Giacomotto J, Segalat L (2010). High-throughput screening and small animal models, where are we?. Br J Pharmacol.

[54] Szewczyk NJ, Kozak E, Conley CA (2003). Chemically defined medium and Caenorhabditis elegans.. BMC Biotechnol.

[55] Ellerbrock BR, Coscarelli EM, Gurney ME, Geary TG (2004). Screening for presenilin inhibitors using the free-living nematode, Caenorhabditis elegans.. J Biomol Screen.

[56] Okoli I, Coleman JJ, Tampakakis E, An WF, Holson E (2009). Identification of antifungal compounds active against Candida albicans using an improved high-throughput Caenorhabditis elegans assay.. PLoS One.

[57] Williams A, Sarkar S, Cuddon P, Ttofi EK, Saiki S (2008). Novel targets for Huntington's disease in an mTOR-independent autophagy pathway.. Nat Chem Biol.

[58] Zhang L, Yu J, Pan H, Hu P, Hao Y (2007). Small molecule regulators of autophagy identified by an image-based high-throughput screen.. Proc Natl Acad Sci U S A.

[59] Kamimoto T, Shoji S, Hidvegi T, Mizushima N, Umebayashi K (2006). Intracellular inclusions containing mutant alpha1-antitrypsin Z are propagated in the absence of autophagic activity.. J Biol Chem.

[60] Link CD (1995). Expression of human beta-amyloid peptide in transgenic Caenorhabditis elegans.. Proc Natl Acad Sci U S A.

[61] Kraemer BC, Zhang B, Leverenz JB, Thomas JH, Trojanowski JQ (2003). Neurodegeneration and defective neurotransmission in a Caenorhabditis elegans model of tauopathy.. Proc Natl Acad Sci U S A.

[62] Lakso M, Vartiainen S, Moilanen AM, Sirvio J, Thomas JH (2003). Dopaminergic neuronal loss and motor deficits in Caenorhabditis elegans overexpressing human alpha-synuclein.. J Neurochem.

[63] Faber PW, Alter JR, MacDonald ME, Hart AC (1999). Polyglutamine-mediated dysfunction and apoptotic death of a Caenorhabditis elegans sensory neuron.. Proc Natl Acad Sci U S A.

[64] Oeda T, Shimohama S, Kitagawa N, Kohno R, Imura T (2001). Oxidative stress causes abnormal accumulation of familial amyotrophic lateral sclerosis-related mutant SOD1 in transgenic Caenorhabditis elegans.. Hum Mol Genet.

[65] Nehrke K, Melvin JE (2002). The NHX family of Na+-H+ exchangers in Caenorhabditis elegans.. J Biol Chem.

[66] Johnston PA, Foster CA, Shun TY, Skoko JJ, Shinde S (2007). Development and implementation of a 384-well homogeneous fluorescence intensity high-throughput screening assay to identify mitogen-activated protein kinase phosphatase-1 dual-specificity protein phosphatase inhibitors.. Assay Drug Dev Technol.

[67] Brideau C, Gunter B, Pikounis B, Liaw A (2003). Improved statistical methods for hit selection in high-throughput screening.. J Biomol Screen.

[68] Malo N, Hanley JA, Cerquozzi S, Pelletier J, Nadon R (2006). Statistical practice in high-throughput screening data analysis.. Nat Biotechnol.

[69] Dantuma NP, Lindsten K, Glas R, Jellne M, Masucci MG (2000). Short-lived green fluorescent proteins for quantifying ubiquitin/proteasome-dependent proteolysis in living cells.. Nat Biotechnol.

