# Supplementary figures and images for: Automated High-Content Live Animal Drug Screening Using C. elegans Expressing the Aggregation Prone Serpin α1-antitrypsin Z
Source: PLoS One. 2010 Nov 12;5(11):e15460. doi: 10.1371/journal.pone.0015460 (PMC2980495; doi:10.1371/journal.pone.0015460)

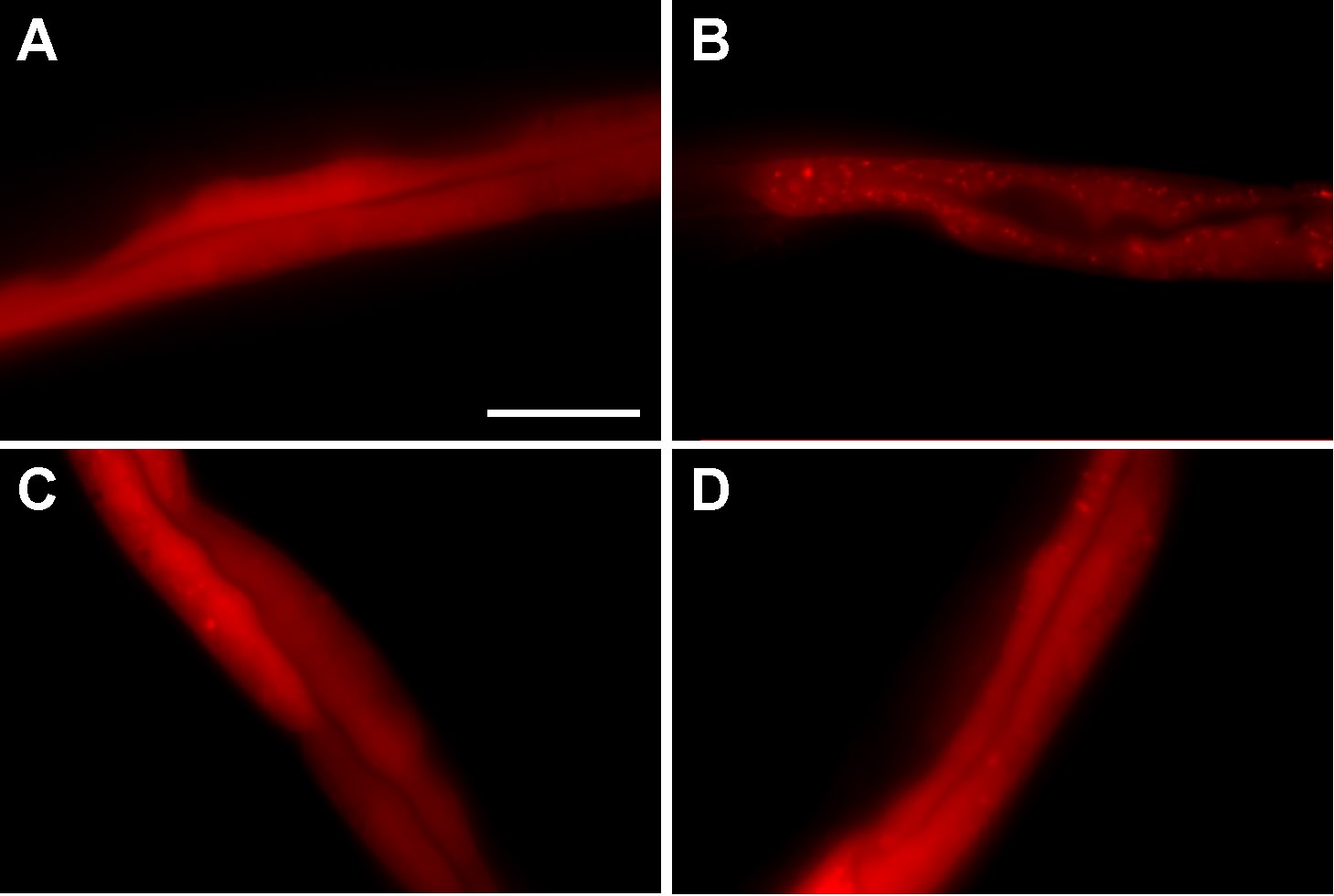

Supplement: Figure S1 — Effect of fluphenazine on mCherry aggregation. To determine whether fluphenazine causes non-specific aggregation of mCherry, transgenic animals expressing Pnhx-2Ub-M::mCherry were exposed to fluphenazine for 24 hours. The Ub-M::mCherry fusion is used as a control for ubiquitin (Ub) fusion protein degradation. Upon Ub cleavage, the absence of a degradation signal (due to an N-terminal methionine) prevents mCherry degradation. Thus, mCherry behaves as an unmodified cytosolic protein [69]. Pnhx-2mCherry::lgg-1 (top) and Pnhx-2Ub(GM)::mCherry (bottom) animals were treated with (A, C) 0.5% DMSO or (B, D) 50 µM fluphenazine. Images were acquired using a Nikon instruments TiEclipse widefield light microscope fitted with a 40× objective. Scale bar, 100 µm. (JPG) [file pone.0015460.s001.jpg]
